# ^18^F-THK5351 PET for visualizing predominant lesions of pathologically confirmed corticobasal degeneration presenting with frontal behavioral-spatial syndrome

**DOI:** 10.1007/s00415-022-11121-y

**Published:** 2022-04-13

**Authors:** Yuji Saitoh, Etsuko Imabayashi, Masashi Mizutani, Tadashi Tsukamoto, Masato Hasegawa, Yuko Saito, Hiroshi Matsuda, Yuji Takahashi

**Affiliations:** 1 Department of Neurology, National Center Hospital, National Center of Neurology and Psychiatry, 4-1-1 Ogawa-Higashi, Kodaira, Tokyo 187-8551 Japan; 2 Research Center for Neurocognitive Disorders, National Center Hospital, National Center of Neurology and Psychiatry, 4-1-1 Ogawa-Higashi, Kodaira, Tokyo 187-8551 Japan; 3 Integrative Brain Imaging Center, National Center of Neurology and Psychiatry, 4-1-1 Ogawa-Higashi, Kodaira, Tokyo 187-8551 Japan; 4 Department of Molecular Imaging and Theranostics, Quantum Life and Medical Science Directorate, National Institute for Quantum Science and Technology, 4-9-1 Anagawa, Inage-ku, Chiba, 263-8555 Japan; 5 Department of Laboratory Medicine, National Center Hospital, National Center of Neurology and Psychiatry, 4-1-1 Ogawa-Higashi, Kodaira, Tokyo 187-8551 Japan; 6 Dementia Research Project, Tokyo Metropolitan Institute of Medical Science, 2-1-6 Kamikitazawa, Setagaya-ku, Tokyo, 156-8506 Japan; 7 Present Address: Department of Neuropathology (Brain Bank for Aging Research), Tokyo Metropolitan Institute of Gerontology, 35-2 Sakaecho, Itabashi-ku, Tokyo, 173-0015 Japan; 8 Present Address: Department of Biofunctional Imaging, Fukushima Medical University, 2-2-1 Otemachi, Chiyoda-ku, Tokyo, 100-0004 Japan

Dear Sirs,

Clinical phenotypes of corticobasal degeneration (CBD) vary and are typically presented with four phenotypes: corticobasal syndrome (CBS), frontal behavioral-spatial syndrome (FBS), nonfluent/agrammatic variant of primary progressive aphasia, and progressive supranuclear palsy syndrome [1]. FBS is the third most common phenotype of pathologically verified CBD, accounting for approximately 14% of CBD patients [1]. Conversely, the pathological features of the clinical phenotype of behavior variant frontotemporal dementia (bvFTD) also vary, and approximately 9% of these patients have been verified pathologically CBD [2]. Here, we present an autopsy-confirmed case of CBD presenting with FBS who underwent positron emission tomography (PET) with ^18^F-THK5351, visualizing the predominant lesion of the frontal lobes associated with the clinical phenotype.

A 72-year-old right-handed male developed gait slowing. Three years later, he lost his way when climbing mountains and was found lying. Two months later, he lost his way again in the neighborhood, and eventually, his wife started accompanying him when he went outside. He developed urinary incontinence, masked face, decreased speech output, visual hallucination, and abnormal behaviors, such as nocturnal wandering, pica, and apraxia. His condition was evaluated at an outpatient neurology clinic, and neurological examination revealed his masked face, bradykinesia, and rigidity of his neck and left-sided upper limb. He showed no therapeutic response to levodopa for parkinsonism. His abnormal behavior increased with time and he showed stereotyped behavior, such as keeping cleaning a room or washing his body. He visited our hospital for further evaluation of his neuropsychiatric symptoms at the age of 75.

Neurological examination revealed left-sided predominant parkinsonism, apraxia, and perseveration. He showed severe cognitive impairment and scored 19/30 on the Mini-Mental State Examination and 3/18 on the Frontal Assessment Battery. Brain magnetic resonance imaging (MRI) at the age of 75 revealed right-sided and frontal lobe dominant atrophy (Fig. 1A,B), which was verified using the voxel-based specific regional analysis system for Alzheimer’s disease [3] (Fig. 1C). Dopamine transporter single-photon emission computed tomography with ^123^I-N-ω-fluoropropyl-2β-carboxymethoxy-3β-(4-iodophenyl)nortropane obtained 4 months before he visited us showed diffusely reduced uptake in the bilateral striata with right-sided predominance (Fig. 1D). He underwent ^18^F-THK5351 PET (Fig. 1E–G), ^18^F-fluorodeoxyglucose (^18^F-FDG) PET (Fig. 1H), and ^11^C-Pittsburgh compound B (^11^C-PiB) PET at the age of 75. The *Z* score map of ^18^F-THK5351 was superimposed on spatially normalized T1-weighted image (Fig. 1G). The *Z* scores were calculated as: (mean voxel value of 30 cognitively unimpaired subjects—patient voxel value)/standard deviation of 30 cognitively unimpaired subjects with cerebellar cortex as reference. ^18^F-THK5351 accumulated in the frontal lobes with right-sided predominance, as well as in the parietal lobes (Fig. 1E–G). Hypometabolism of both the frontal and parietal lobes with right-sided predominance was detected by ^18^F-FDG PET (Fig. 1H). Amyloid deposition was not identified by ^11^C-PiB PET (data not shown). He was clinically diagnosed with bvFTD underlying tau-pathology, that is, frontotemporal lobar degeneration tau, according to the prominent frontal symptoms with spatial impairment, parkinsonism, and neuroimaging findings including ^18^F-THK5351 PET. He died of aspiration pneumonia at the age of 76.

**Fig. 1:**
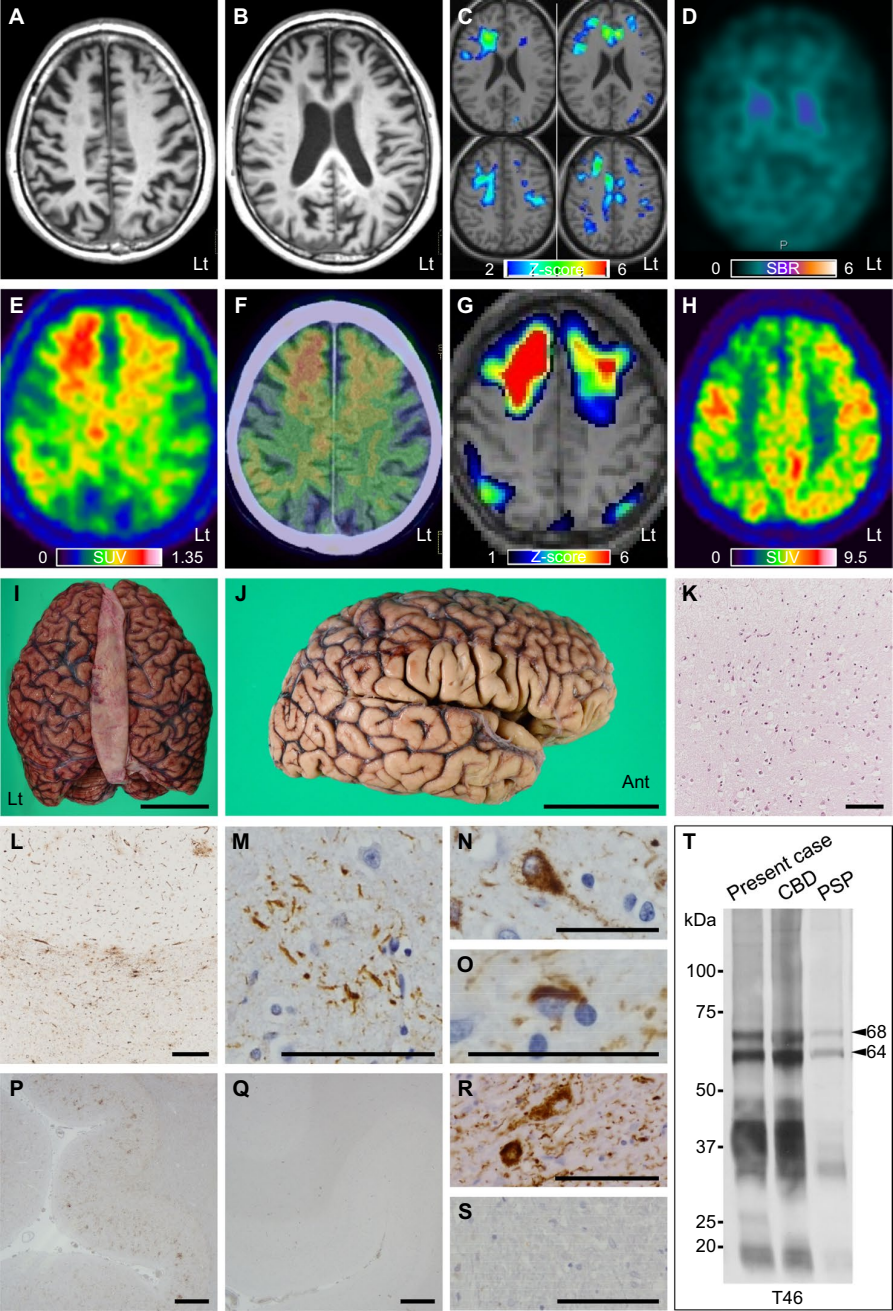
Neuroradiological and histopathological findings and western blot analysis. **A–C** Brain magnetic resonance imaging demonstrates right-sided and frontal predominant atrophy (**A**, **B**). The voxel-based specific regional analysis system for Alzheimer’s disease reveals the right-sided predominant atrophy both in white matter (**C**, left column) and in gray matter (**C**, right column), being 2 standard deviations lower than the average volume of cognitively unimpaired elderly. **D** Dopamine transporter SPECT with ^123^I-FP-CIT shows diffusely reduced uptake in the bilateral striata with right-sided predominance. **E–G** ^18^F-THK5351 PET demonstrates abnormal accumulation in the frontal lobes with right-sided predominance, as well as the parietal lobes. ^18^F-THK5351 PET image was superimposed on brain computed tomography of the patient (**F**). *Z* score of ^18^F-THK5351 compared to 30 cognitively unimpaired subjects as control was superimposed on spatially normalized T1-weighted image (**G**). **H** ^18^F-fluorodeoxyglucose PET shows hypometabolism of both the frontal and parietal lobes with right-sided predominance. **I**, **J** The macroscopic appearance of the whole brain and right brain. There is cerebral atrophy of the frontal and temporal lobes (**I**). There is dilation of the Sylvian fissure and mild atrophy of the frontal operculum in the right brain (**J**). **K**, **L** In the right frontal lobe, hematoxylin–eosin staining shows rarefaction of tissue (**K**), and immunohistochemistry using anti-vimentin antibody reveals vimentin-immunoreactive astrocytes along with the corticomedullary junction, reflecting astrogliosis (**L**). **M–O** Immunohistochemistry of frontal lobes using AT8 antibody reveals phosphorylated tau-positive astrocytic plaques (**M**), pretangles (**N**), and coiled bodies (**O**), accompanying tau-positive threads. **P, Q** Immunoreactivity for AT8 antibody in frontal lobes is predominant on the right side (**P**) compared to the left side (**Q**). **R**, **S** Immunohistochemistry shows that tau-positive deposition is immunoreactive for anti-4R (RD4) (**R**), but not for anti-3R (RD3) (**S**). **T** Western blot analysis of sarkosyl-insoluble tau from the brain probed by T46 antibody shows a major doublet of 68 and 64 kDa, which corresponds to hyperphosphorylated full-length 4-repeat tau isoforms. Note the prominent C-terminal fragments of tau with ~ 37 kDa of this case are similar to those of CBD, but not to PSP. Bars, 5 cm (**I**, **J**), 100 µm (**K–M**, **R**, **S**), 50 µm (**N**, **O**), 1 mm (**P**, **Q**). *CBD* corticobasal degeneration, ^*123*^*I-FP-CIT* ^123^I-N-ω-fluoropropyl-2β-carboxymethoxy-3β-(4-iodophenyl)nortropane, *PET* positron emission tomography, *PSP* progressive supranuclear palsy, *SBR* specific binding ratio, *SPECT* single-photon emission computed tomography, *SUV* standardized uptake value

An autopsy was performed after consent was obtained from his family. The brain weighed 1402 g and showed cerebral atrophy, especially of the frontal and temporal lobes (Fig. 1I). There was dilation of the Sylvian fissure and mild atrophy of the frontal operculum with right-sided predominance (Fig. 1J). Microscopic assessment showed neuronal loss and an increased number of astrocytes in the cerebral cortex, especially in the frontal lobes, accompanied by abnormal rarefaction of tissue (Fig. 1K, L). Immunohistochemistry of the frontal lobes using AT8 antibody revealed phosphorylated tau-positive astrocytic plaques, pretangles, coiled bodies, and threads (Fig. 1M–O), which were presented as right-sided predominance (Fig. 1P, Q). Staining for RD4 or RD3 revealed phosphorylated 4-repeat but not 3-repeat tau-positivity (Fig. 1R, S). These tau-related pathological characteristics in the cortex were prominent in the anterior part of the frontal lobes with right-sided predominance. These were also found in the substantia nigra, subthalamic nucleus, thalamus, globus pallidus, putamen, nucleus basalis of Meynert, locus coeruleus, and inferior olivary nucleus. Western blot analysis of sarkosyl-insoluble tau from the brain showed a major doublet of 68 and 64 kDa with predominant ~ 37 kDa fragments (Fig. 1T) [4]. These pathological and biochemical findings were consistent with CBD. He was finally diagnosed with CBD-FBS [1].

Various tau PET tracers are under rapid development to visualize abnormal tau accumulation for both diagnosing tauopathy and evaluating the therapeutic effects of new drugs against tauopathy. ^18^F-THK5351, a first-generation tau tracer, was originally developed to detect abnormal tau-pathology [5]. However, recent studies revealed that ^18^F-THK5351 binds to monoamine oxidase-B (MAO-B) highly expressed in astrocytes as off-target binding [6, 7]. Using the binding affinity to MAO-B, ^18^F-THK5351 visualizes the astrogliosis reflecting neurodegenerative changes in various neurological diseases other than tauopathy, such as amyotrophic lateral sclerosis [8].

The main clinical phenotypes of our case imply that the core lesions were in the frontal lobes. Conventional morphological and functional imaging modalities using brain MRI and ^18^F-FDG PET imaging showed both atrophy and hypometabolism with right-sided predominance, respectively. Being consistent with these results of the imaging study, ^18^F-THK5351 PET accumulated in similar lesions, which concurred with the previous study [9]. In addition, these abnormalities obtained from in vivo imaging studies were verified through pathological evaluation of tau-positive deposition and astrogliosis in the frontal lobes with right-sided predominance. Despite the neuropathological features of bvFTD being heterogeneous [2], previous studies on ^18^F-THK5351 PET with bvFTD patients lack pathological confirmation. To our knowledge, this is the first clinicopathological case report demonstrating ^18^F-THK5351 accumulation in the frontal lobes in a CBD-FBS patient, in which the presence of tau-related neurodegenerative change was pathologically verified. However, our study was unable to distinguish the ^18^F-THK5351 accumulation derived from tau accumulation, an increased number of MAO-B positive astrocytes, or both, because of the limited discriminability of ^18^F-THK5351 between tau and MAO-B. Thus, pathologically, radiologically, and biochemically validated MAO-B PET tracers, which can more sensitively visualize neurodegeneration consisting of astrogliosis, are required [10]. Our findings provide the use of ^18^F-THK5351 PET as a marker closely associated with tau-related neurodegeneration.

In conclusion, the ^18^F-THK5351 PET image visualizes abnormal tau-related neurodegeneration reflecting clinicopathological severities in CBD-FBS. Our case highlights that ^18^F-THK5351 PET can be a potent technique for visualizing the tau-related predominant lesions of CBD-FBS and for discriminating the underlying pathologies of bvFTD.

## Abbreviations

bvFTD: Behavior variant frontotemporal dementia
^11^C-PiB: ^11^C-Pittsburgh compound B
CBD: Corticobasal degeneration
DaT: Dopamine transporter
^18^F-FDG: ^18^F-fluorodeoxyglucose
FBS: Frontal behavioral-spatial syndrome
^123^I-FP-CIT: ^123^I-N-ω-fluoropropyl-2β-carboxymethoxy-3β-(4-iodophenyl)nortropane
MAO-B: Monoamine oxidase-B
MRI: Magnetic resonance imaging
PET: Positron emission tomography
PSP: Progressive supranuclear palsy
SBR: Specific binding ratio
SPECT: Single-photon emission computed tomography
SUV: Standardized uptake value
